# Relationship between peripheral airway function and patient-reported outcomes in COPD: a cross-sectional study

**DOI:** 10.1186/1471-2466-10-10

**Published:** 2010-03-07

**Authors:** Akane Haruna, Toru Oga, Shigeo Muro, Tadashi Ohara, Susumu Sato, Satoshi Marumo, Daisuke Kinose, Kunihiko Terada, Michiyoshi Nishioka, Emiko Ogawa, Yuma Hoshino, Toyohiro Hirai, Kazuo Chin, Michiaki Mishima

**Affiliations:** 1 Department of Respiratory Medicine, Graduate School of Medicine, Kyoto University, Kyoto, Japan; 2 Department of Respiratory Care and Sleep Control Medicine, Graduate School of Medicine, Kyoto University, Kyoto, Japan

## Abstract

**Background:**

Health status, dyspnea and psychological status are important clinical outcomes in chronic obstructive pulmonary disease (COPD). However, forced expiratory volume in one second (FEV_1_) measured by spirometry, the standard measurement of airflow limitation, has only a weak relationship with these outcomes in COPD. Recently, in addition to spirometry, impulse oscillometry (IOS) measuring lung resistance (R) and reactance (X) is increasingly being used to assess pulmonary functional impairment.

**Methods:**

We aimed to identify relationships between IOS measurements and patient-reported outcomes in 65 outpatients with stable COPD. We performed pulmonary function testing, IOS, high-resolution computed tomography (CT), and assessment of health status using the St. George's Respiratory Questionnaire (SGRQ), dyspnea using the Medical Research Council (MRC) scale and psychological status using the Hospital Anxiety and Depression Scale (HADS). We then investigated the relationships between these parameters. For the IOS measurements, we used lung resistance at 5 and 20 Hz (R5 and R20, respectively) and reactance at 5 Hz (X5). Because R5 and R20 are regarded as reflecting total and proximal airway resistance, respectively, the fall in resistance from R5 to R20 (R5-R20) was used as a surrogate for the resistance of peripheral airways. X5 was also considered to represent peripheral airway abnormalities.

**Results:**

R5-R20 and X5 were significantly correlated with the SGRQ and the MRC. These correlation coefficients were greater than when using other objective measurements of pulmonary function, R20 on the IOS and CT instead of R5-R20 and X5. Multiple regression analyses showed that R5-R20 or X5 most significantly accounted for the SGRQ and MRC scores.

**Conclusions:**

IOS measurements, especially indices of peripheral airway function, are significantly correlated with health status and dyspnea in patients with COPD. Therefore, in addition to its simplicity and non-invasiveness, IOS may be a useful clinical tool not only for detecting pulmonary functional impairment, but also to some extent at least estimating the patient's quality of daily life and well-being.

## Background

Health status, dyspnea and psychological status of anxiety and depression are important outcomes in chronic obstructive pulmonary disease (COPD). Improved health status and relief of symptoms are listed as the goals of effective management in the Global Initiative for Chronic Obstructive Lung Disease (GOLD) [1].

Mortality is also an important outcome in COPD as it is ranked as high as a cause of death in the world [1]. The above-mentioned patient-reported outcomes can significantly predict mortality independently of airflow limitation evaluated by forced expiratory volume in one second (FEV_1_) [2-4]. In fact, previous reports indicate that there are only weak correlations between these outcomes and FEV_1 _[5-8].

Recent studies suggest that impulse oscillometry (IOS) can detect airway abnormalities better than spirometry for assessing pulmonary function in COPD [9,10], adult asthma [11-13], and dust exposure [14,15]. IOS measures lung resistance and reactance, and is a clinically promising method which is simple and non-invasive and does not require forced maneuvers [16,17].

Comparing FEV_1 _by spirometry and IOS, while FEV_1 _mainly measures expiratory flow at high and mid lung volumes [18], IOS can usefully measure both large and small airway resistance separately [14-17]. This feature of IOS may be important because the involvement of small airways in COPD is indicated [19-21].

In assessing COPD, high-resolution computed tomography (CT) also provides valuable information concerning parenchymal destruction and airway lesions. We have assessed the severity of emphysema by measuring the low attenuation area (LAA), and airway dimensions including evaluation of the luminal area and airway wall thickness of the right upper apical bronchus [22].

We hypothesized that there would be significant associations between patient- reported outcomes and pulmonary impairments evaluated with IOS in patients with COPD. Here, we performed a cross-sectional study evaluating three different objective assessments consisting of conventional pulmonary function tests, IOS and CT, and compared them with subjective measurements of health status, dyspnea and psychological status in patients with COPD.

## Methods

### Subjects

We recruited 65 male outpatients with stable COPD, defined by the GOLD [1], at Kyoto University Hospital. The eligibility criteria included: 1) smoking history of more than 20 pack-years; 2) maximal FEV_1_/forced vital capacity (FVC) ratio of less than 0.7; 3) regular management and treatment at our outpatient clinic over 6 months; 4) no exacerbations in the previous 6 weeks; 5) no other lung diseases; 6) no uncontrolled comorbidities such as severe cardiovascular diseases and malignant disorders; and 7) having sufficient cognitive function to complete the questionnaire. Pulmonary function, IOS, CT, health status, dyspnea, and psychological status were assessed on the same day. The research protocol was approved by the Ethics Committee of Kyoto University and the subjects gave written informed consent.

### Outcome measures

#### Pulmonary function

Spirometry was performed according to the recommended method, as reported previously [2,6,7]. Residual volume (RV) and total lung capacity (TLC) were measured by the closed-circuit helium method, and carbon monoxide diffusing capacity (DL_CO_) was measured using a single-breath technique.

#### IOS

The impedance of the total respiratory system was measured using IOS (Erich Jaeger, Germany). Subjects underwent IOS and then spirometry 15 minutes after inhaling the bronchodilators salbutamol (400 μg) and ipratropium bromide (80 μg) [2,6,7]. The superimposed pressure oscillations during normal-volume spontaneous breathing are composed of several frequencies, allowing the assessment of resistance (R) and reactance (X) at several frequencies simultaneously. The frequency range of the signal was from 5 to 35 Hz. Subjects supported their cheeks to reduce upper airway shunting while impulses were applied during tidal breathing for 30 seconds. In the present study, we used respiratory resistance at 5 and 20 Hz (R5 and R20, respectively) and reactance at 5 Hz (X5) for the analyses. R5 and R20 are regarded as reflecting total and proximal airway resistance, respectively, and the fall in resistance from R5 to R20 (R5-R20) was used as a surrogate for the resistance of peripheral airways, as reported previously [14-17,23-27]. Furthermore, X5, which may be determined by homogeneous distribution of ventilation, effective ventilation capacity, and compliance of the lung and chest wall, was also considered to represent peripheral airway abnormalities such as those caused by inflammation [15-17,23-25]. X5 was recently reported to be a useful and informative measurement due to its close relationship with conventional pulmonary function assessments in COPD [28].

#### CT

CT images were obtained using a multi-detector row scanner (Aquilion 64; Toshiba, Tokyo, Japan) with simultaneous acquisition of sixty-four 0.5 mm sections. Technical parameters were as follows: voltage 120 kVp, automatic electric current control, field of view 350 mm, reconstruction algorithm, lung algorithm with automatic correction of beam hardening effect. Percentages of LAA (LAA%) were analyzed according to a modified method that was previously reported from our department [29]. Unlike the original method, in the current study, we used multi-detector computed tomography (MDCT) that provided us with many slices, but without additional radiation exposure. Therefore, we used the slices from the upper margin of the aortic arch to the diaphragm to analyze LAA%. Areas where CT numbers were less than -200 HU were defined as lung fields, and the cut-off level between LAA and normal lung density was defined as -960 HU [22,30].

For the analysis of airway dimensions, the right upper apical bronchus was measured in each subject using the method we had previously reported [22,30,31]. The area of the inner lumen and thickness of the airway wall was measured automatically from CT images. The diameter of the airway, the outer area of the airway, airway wall area (WA), and percentage wall area (WA%) were calculated from the area of the inner lumen and thickness of the airway wall.

#### Health status

Health status was evaluated by the Japanese version of the St. George's Respiratory Questionnaire (SGRQ) [2,6,7,32]. It contains 50 items divided into 3 components of symptoms, activity and impacts, and a total score was also calculated, with scores ranging from 0 to 100. Lower scores indicate less impairment on the SGRQ.

#### Dyspnea

Severity of dyspnea was evaluated by the Japanese version of the modified Medical Research Council (MRC) scale [6,7,33], which consists of a 5-point scale based on degrees of various physical activities that precipitate dyspnea. Lower scores indicate less impairment on the MRC scale.

#### Psychological status

Psychological status was evaluated by the Japanese version of the Hospital Anxiety and Depression Scale (HADS) [6,7,34] which contains 14 items and consists of two subscales: anxiety and depression. Each item is rated on a four-point scale (0-3) where a score of 3 represents the worst state of anxiety or depression.

### Statistical analysis

Results are expressed as mean ± SD. Relationships between different objective measurements (pulmonary function testing, IOS and CT) and patient-reported outcomes were evaluated using Pearson's correlation coefficient tests. We have chosen to present all results as Pearson's correlation coefficients for ease of comprehension and comparison across relationships [35]. We also tested for significant differences between two correlation coefficients obtained from two different relationships in the same samples [36]. Next, we performed forward and backward stepwise multiple regression analyses to identify the variables that could best predict the health status and dyspnea scores, using the measurements of pulmonary function, IOS and CT as independent variables [6]. P values of less than 0.05 were considered to be statistically significant. All analyses were performed using the JMP (SAS Institute).

## Results

### Baseline characteristics

Baseline characteristics of the 65 male patients with COPD are presented in Table 1. Of these, 11 (17.0%) were current smokers. COPD severity based on the GOLD [1] was mild in 12 patients (18.4%), moderate in 30 (46.2%), severe in 20 (30.8%), and very severe in the remaining 3 patients (4.6%).

**Table 1:** Baseline characteristics (n = 65)

Characteristics	Mean ± SD	Range
Age (years)	71 ± 9	46 -- 87
Height (m)	1.65 ± 0.06	1.50 -- 1.80
Body mass index (kg/m^2^)	21.3 ± 2.7	15.42 -- 29.0
FEV_1 _(L)	1.64 ± 0.64	0.58 -- 3.42
FEV_1 _(%predicted)	58.8 ± 19.7	18.7 -- 100.8
RV/TLC (%)	42.9 ± 7.5	27.7 -- 59.4
DL_CO_/V_A_(ml/min/mmHg/L)	2.76 ± 1.06	0.69 -- 5.35
R20 (kPa/L/s)	0.24 ± 0.07	0.13 -- 0.41
R5-R20 (kPa/L/s)	0.09 ± 0.06	-0.02 -- 0.27
X5 (kPa/L/s)	-0.15 ± 0.08	-0.48 -- -0.05
LAA (%)	20.4 ± 10.2	2.5 -- 44.5
WA (%)	57.1 ± 5.7	44.9 -- 71.6
SGRQ symptoms (0--100)	35.0 ± 20.5	0 -- 90.6
SGRQ activity (0--100)	41.2 ± 21.9	0 -- 92.5
SGRQ impact (0--100)	20.1 ± 15.9	0 -- 63.2
SGRQ total (0--100)	29.0 ± 16.4	1.6 -- 62.3
MRC (0--4)	1.1 ± 0.8	0 -- 4
HADS anxiety (0--21)	3.3 ± 3.0	0 -- 12
HADS depression (0--21)	3.6 ± 2.9	0 -- 12

Numbers in parentheses indicate theoretical score ranges.

FEV_1 _= forced expiratory volume in one second; RV = residual volume; TLC = total lung capacity; DL_CO _= carbon monoxide diffusing capacity; V_A _= alveolar volume R = resistance; X = reactance; LAA = low attenuation area; WA = wall area; SGRQ = St. George's Respiratory Questionnaire; MRC = Medical Research Council; HADS = Hospital Anxiety and Depression Scale

### Relationships between IOS measurements, pulmonary function and CT

We examined the relationships between IOS measurements and other objective parameters of pulmonary function and CT. R20 showed a weak or non-significant correlation with R5-R20 and X5 (*r *(correlation coefficient) = 0.29, *p *= 0.020, and *r *= -0.19, *p *= 0.13, respectively), indicating R20 reflects different physiological parameters from R5-R20 and X5. In contrast, there was a strong association between R5-R20 and X5 (*r *= -0.87, *p *< 0.001), indicating that these two peripheral airway functional indices could reflect similar events. R20 was significantly but weakly correlated with FEV_1 _(*r *= -0.29, *p *= 0.018). R5-R20 and X5 were moderately significantly correlated with FEV_1 _(*r *= -0.69, *p *< 0.001 and *r *= 0.61, *p *< 0.001, respectively) and RV/TLC (*r *= 0.49, *p *< 0.001 and *r *= -0.54, *p *< 0.001, respectively) and weakly with LAA% (*r *= 0.32, *p *= 0.010 and *r *= -0.37, *p *= 0.002, respectively).

### Relationships between pulmonary function, IOS, CT and patient-reported outcomes

Table 2 shows the correlation coefficients for pulmonary function, IOS, CT and patient-reported outcomes. R5-R20 and X5 on IOS were weakly to moderately correlated with all three components (symptoms, activity and impacts) and the total of the SGRQ and the MRC scores (*r *= 0.31-0.51, *p *< 0.01 and *r *= 0.27-0.51, *p *< 0.01, respectively). These relationships were significantly stronger than any other relationships for each component and total of the SGRQ and MRC scores [36].

**Table 2:** Analysis of the relationships between pulmonary function, IOS, CT, and patient-reported outcomes

	SGRQ				MRC	HADS	
	symptom	activity	impacts	total		anxiety	depression
FEV_1_	-0.24	-0.41	-0.29	-0.36	-0.37	--	--
RV/TLC	--	0.40	--	0.29	0.46	--	--
DL_CO_/V_A_	--	--	--	--	--	--	--
R20	--	--	--	--	--	--	--
R5-R20	0.31	0.49	0.44	0.49	0.51	--	--
X5	-0.27	-0.51	-0.38	-0.46	-0.46	--	--
LAA%	--	0.27	--	--	0.41	--	--
WA%	--	--	--	--	--	--	--

All values represent correlation coefficient (r).

Missing data (--) indicate that there were no statistically significant relationships.

FEV_1 _= forced expiratory volume in one second; RV = residual volume; TLC = total lung capacity; DL_CO _= carbon monoxide diffusing capacity; V_A _= alveolar volume R = resistance; X = reactance; LAA = low attenuation area; WA = wall area; SGRQ = St. George's Respiratory Questionnaire; MRC = Medical Research Council; HADS = Hospital Anxiety and Depression Scale

### Results of multiple regression analyses to predict health status and dyspnea

**Figure 1:**
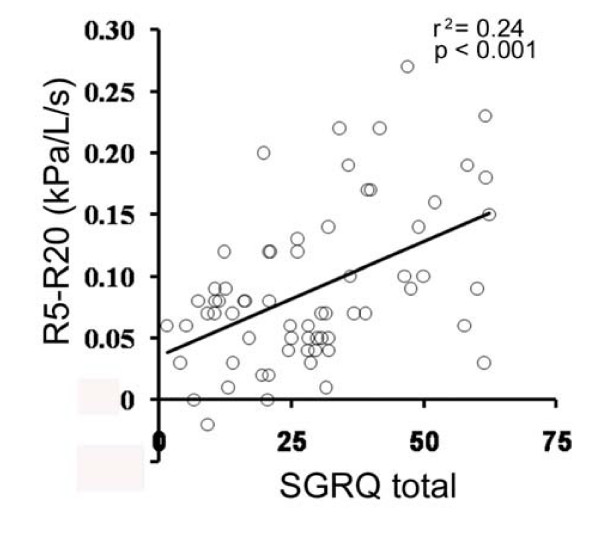
**Relationship between R5-R20 and SGRQ total score**. R5-R20 showed a significant positive correlation with SGRQ total score (*r*^2 ^= 0.24, *p *< 0.001).

Table 3 shows the results of stepwise multiple regression analyses to identify variables that could best predict health status and dyspnea. R5-R20 alone accounted for the symptoms, impacts and total of the SGRQ significantly (*r*^2 ^(coefficient of determination) = 0.09, 0.19 and 0.24, respectively). The relationship between R5-R20 and the total SGRQ is shown in Figure 1. The variance of the activity of the SGRQ was most significantly accounted for by X5 (*r*^2 ^= 0.26), and far less so by RV/TLC (*r*^2 ^= 0.02). For the MRC scores, R5-R20 was the most significant contributive factor (*r*^2 ^= 0.27), and LAA%, FEV_1_, and RV/TLC were found to be only weakly associated (*r*^2 ^= 0.06, 0.03 and 0.02, respectively).

**Table 3:** Analysis of the relationships of health status and dyspnea to pulmonary function, IOS and CT by stepwise multiple regression analyses

	SGRQ				
	symptoms	activity	impacts	total	MRC
Independent variables					
FEV_1_	--	--	--	--	0.03
RV/TLC	--	0.02	--	--	0.02
R5-R20	0.09	--	0.19	0.24	0.27
X5	--	0.26	--	--	--
LAA%	--	--	--	--	0.06
Cumulative r^2^	0.09	0.28	0.19	0.24	0.38

All values represent coefficient of determination (r^2^).

Missing data (--) indicate that independent variables were not statistically significant.

FEV_1 _= forced expiratory volume in one second; RV = residual volume; TLC = total lung capacity; R = resistance; X = reactance; LAA = low attenuation area; SGRQ = St. George's Respiratory Questionnaire; MRC = Medical Research Council

## Discussion

Here, we demonstrated that peripheral airway measurements by IOS (R5-R20 and X5) were significantly correlated with the SGRQ and MRC scores. No measurements of pulmonary function, IOS and CT were significantly correlated with the HADS. Multiple regression analyses revealed that R5-R20 or X5 accounted for the SGRQ and MRC scores more significantly than FEV_1 _and CT measurements (*r*^2 ^= 0.09 to 0.27). Thus, the novel finding in this study is that peripheral airway measurements by IOS were useful not only for assessing pulmonary impairments but also because they have certain relationships with health status and dyspnea in patients with COPD. IOS is easy to perform, only requiring tidal breathing, not forced maneuvers as in conventional pulmonary testing such as spirometry. Therefore, IOS might be considered to be more useful in routine practice for elderly patients with COPD. In fact, IOS was recently used for detecting airway abnormalities and for assessing pharmacological therapeutic efficacy in COPD and other respiratory disorders [11-15,23-27].

Small airway disease (obstructive bronchiolitis) is one of the central pathophysiological features of COPD [1]. Peripheral airway obstruction progressively traps air during expiration, resulting in hyperinflation in COPD [1]. In the present study, peripheral airway measurements (R5-R20 and X5) by IOS consistently correlated with both the degree of airflow limitation (FEV_1_) and the degree of hyperinflation (RV/TLC) due to peripheral airway obstruction. In contrast, large airway function (R20) by IOS was not or only weakly correlated with the degrees of airflow limitation and hyperinflation, and not with health status and dyspnea. In COPD, it is now considered that hyperinflation rather than airflow limitation is the main mechanism for exertional dyspnea [37], and dyspnea is the major determinant of health status [5,6]. The significant association between hyperinflation and patient-reported outcomes of dyspnea and poor health status in patients with COPD has recently been reviewed [38]. Thus, the present study indicates that the peripheral airway may be important in determining pulmonary impairment as well as health status and dyspnea to some extent in patients with COPD, which can easily be assessed using IOS.

CT is also a diagnostic useful method for patients with COPD because it can easily quantitatively assess airway and parenchymal pathology. First, we evaluated WA%, an index of wall thickening in the large airway, to assess airway function [20,32,39]. This parameter can also be used in asthma for correlating airway parameters with disease severity [31]. However, in the present study, WA% was found not to correlate significantly with the SGRQ and MRC scores, indicating that large airway structural alterations on CT may not accurately reflect patients' perception of functional impairment. Secondly, we used LAA% on CT as a quantitative index of emphysema, another key feature of COPD. There are some publications [40-42] on the relationship between emphysema and the severity of COPD. However, multiple regression analyses revealed that LAA% did not or only weakly accounted for the SGRQ and MRC scores, independent of R5-R20 or X5, indicating that emphysema might not be as strong a determinant of health status or dyspnea in COPD as peripheral airway measurements by IOS.

Disturbed psychological status with anxiety and depression is well-known in COPD. Previous studies indicate that it is not significantly correlated with physiological measurements such as FEV_1_, arterial oxygen pressure, exercise intolerance or daily physical activities [4,8,43,44]. Additionally, we show here that, none of the measurements of pulmonary function, IOS or CT was significantly correlated with the HADS. This indicates that psychological status should be evaluated separately from airflow limitation, airway resistance and emphysema.

The values for the coefficient determinations were relatively small in Table 3 (cumulative *r*^2 ^= 0.09 to 0.38), indicating that other factors might be contributing to the outcome. In the present study, we did not assess systemic consequences of COPD, such as exercise capacity and inflammatory biomarkers. These are known to be significantly correlated with health status or dyspnea independently FEV_1 _[5-7,45]. As COPD is regarded as a systemic disease [1], in future, a more comprehensive approach including these parameters might be informative for better understandings the systemic effects of COPD compared to airway diseases.

There are some limitations in this study. First, we used the actual values for IOS for the analysis, as definitive predictive equations have not been established yet [28]. There is a need to perform a large-scale study across a wider age range to validate existing reference values. Second, this study included only male patients, as there are relatively few female patients. This may be due to the gender difference in past smoking trends in Japan, and possibly reflects the present features of Japanese COPD patients. Thus, the generalization of the results to females is unwarranted. Third, the number of the patients included in this study was relatively small. However, our results have generated an interesting hypothesis that small airways disease results in certain symptomatology and this must be tested on new cohorts of patients in order to validate the hypothesis.

## Conclusions

In summary, measurements by IOS are significantly correlated with health status and dyspnea in patients with COPD. Therefore, IOS might be useful to manage male COPD patients assessing also their quality of daily life and well-being.

## Abbreviations

COPD: chronic obstructive pulmonary disease; DL_CO_: carbon monoxide diffusing capacity; FEV_1_: forced expiratory volume in one second; GOLD: Global Initiative for Chronic Obstructive Lung Disease; HADS: Hospital Anxiety and Depression Scale; HRCT: high-resolution computed tomography; IOS: impulse oscillometry; LAA: low attenuation area; MRC: Medical Research Council; R: resistance; RV: residual volume; SGRQ: St. George's Respiratory Questionnaire; TLC: total lung capacity; V_A_: alveolar volume; WA: wall area; X: reactance.

## Competing interests

The authors declare that they have no competing interests.

## Authors' contributions

AH, TO and SM contributed to study design and data acquisition, analysis and interpretation, and prepared the manuscript. TO, SS, SM, DK, KT, MN, EO and YM contributed to data acquisition and critical reviewing of the manuscript. TH, KC and MM supervised the study. All authors read and approved the final manuscript.

## Pre-publication history

The pre-publication history for this paper can be accessed here:

http://www.biomedcentral.com/1471-2466/10/10/prepub
